# Eco-morphodynamic carbon pumping by the largest rivers in the Neotropics

**DOI:** 10.1038/s41598-023-32511-w

**Published:** 2023-04-05

**Authors:** Luca Salerno, Paolo Vezza, Paolo Perona, Carlo Camporeale

**Affiliations:** 1 Department of Environment, Land and Infrastructure Engineering, Politecnico di Torino, Corso Duca degli Abruzzi, Turin, 10129 Italy; 2 Platform of Hydraulic Constructions PL-LCH, Institute of Civil Engineering (IIC), School of Architecture, Civil and Environmental Engineering (ENAC), EPFL, Lausanne, Switzerland

**Keywords:** Carbon cycle, Hydrology, Environmental impact, Geomorphology, Riparian ecology

## Abstract

The eco-morphodynamic activity of large tropical rivers in South and Central America is analyzed to quantify the carbon flux from riparian vegetation to inland waters. We carried out a multi-temporal analysis of satellite data for all the largest rivers in the Neotropics (i.e, width > 200 m) in the period 2000–2019, at 30 m spatial resolution. We developed a quantification of a highly efficient Carbon Pump mechanism. River morphodynamics is shown to drive carbon export from the riparian zone and to promote net primary production by an integrated process through floodplain rejuvenation and colonization. This pumping mechanism alone is shown to account for 8.9 million tons/year of carbon mobilization in these tropical rivers. We identify signatures of the fluvial eco-morphological activity that provide proxies for the carbon mobilization capability associated with river activity. We discuss river migration—carbon mobilization nexus and effects on the carbon intensity of planned hydroelectric dams in the Neotropics. We recommend that future carbon-oriented water policies on these rivers include a similar analysis.

## Introduction

Rivers are not simply passive and static conveyance systems that deliver water and sediments from the headwaters to the oceans, but instead, they actively affect the global carbon budget^1,2^. Although the carbon lateral export from terrestrial ecosystems is recognized to be a key pathway in the biogeochemical carbon cycle^3^, the quantification of carbon mobilization by river dynamics has generally been overlooked^4–7^. By exploring the sediment load—river dynamics—carbon flux nexus of tropical regions of America, we show that river morphodynamics is central to carbon fluxes between terrestrial systems, river corridors and the atmosphere.

Through a global-scale assessment of the dynamics and vegetation density within the Aquatic-Terrestrial Transitional Zone (ATTZ), we demonstrate that the largest tropical rivers in the Neotropics annually recruit 8.90 ± 0.84 million tons of carbon as biomass from live woody riparian vegetation. Through the exploration of an eco-morphodynamic-Carbon-Pumping mechanism, we identify that this recruitment may promote a virtuous cycle for carbon sink, mostly deposited in floodplains but probably even farthest, in oceans.

Under the classical view of the River Continuum Concept^8^, the coarse particulate organic matter exported from floodplains is fragmented and decomposed as it moves downstream, with the consequent transformation into a Particulate and Dissolved Organic Matter (POM and DOM respectively), and then outgassing. However, the fate of LWD recruited by stream waters is far from being fully explained. For example, rivers with high sediment loads have been demonstrated to easily bury wood at least at the same rate as the wood exported to estuaries^9^. Several studies have provided evidence that, once recruited by the channel, LWD can persist buried in the alluvium for extraordinarily long times^10,11^. This suggests that some processes are overlooked in river carbon budgeting^7^. Indeed, riverine sediment storage is a key aspect of biogeochemical cycling^12^, since part of bio-spheric organic carbon is stored in terrestrial reservoirs over millennial timescales before reaching ultimate depocenters in marine basins^13^.

**Figure 1:**
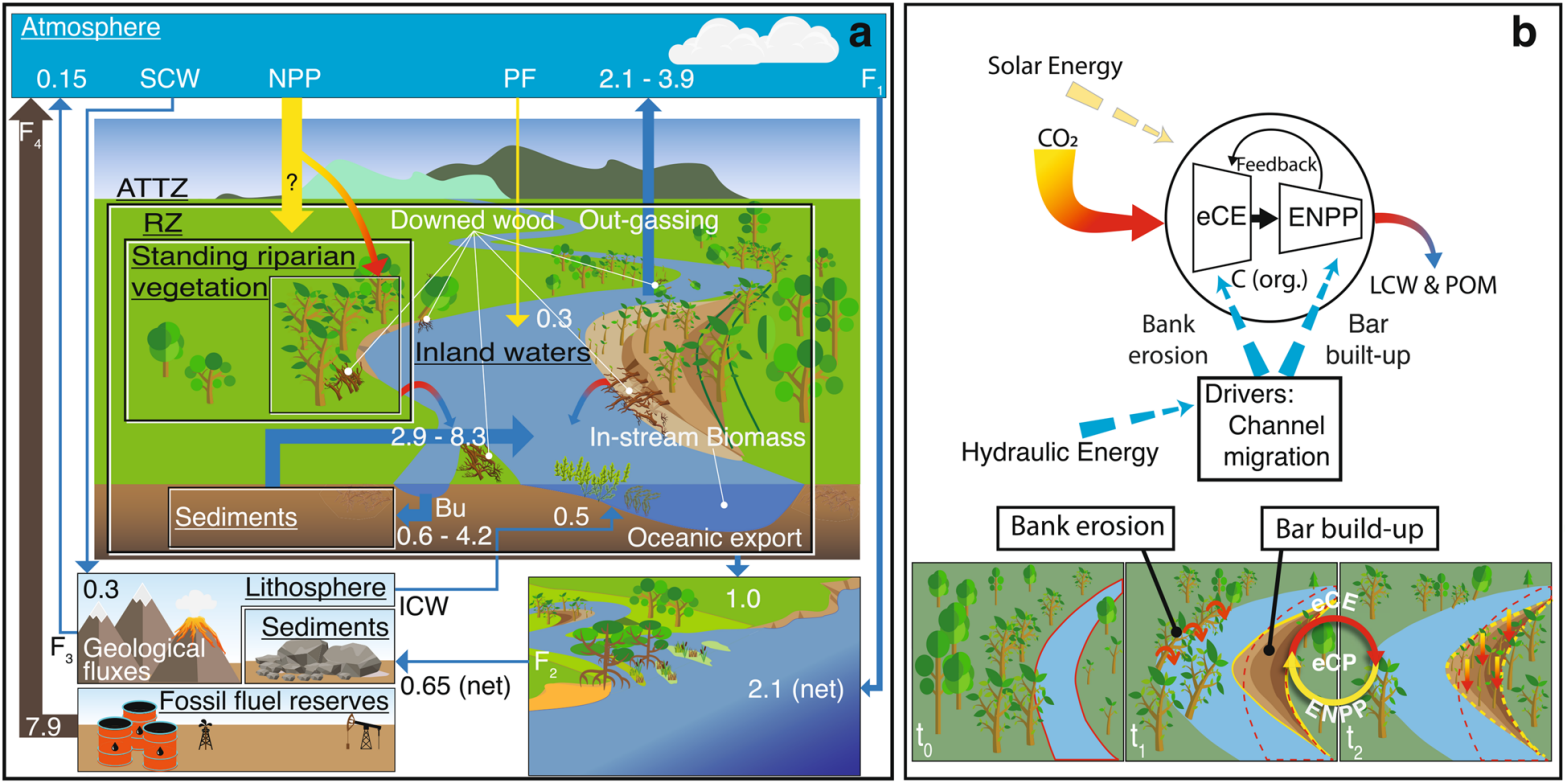
The eco-morphodynamic Carbon Pumping mechanism and global carbon budget of the aquatic-terrestrial transitional zone in rivers, with fluxes reported in PgC/year (global esteems, not only tropical). (**a**) Red-to-blue arrows represent woody vegetation recruited through river morphodynamic activities (eCE), estimated in the present work to 8.9 TgC/year for large tropical rivers (width > 200 m) in the Neotropics (i.e., South and Central America). Yellow-to-red arrows refer to ENPP (see main text). SCW: atmospheric $\hbox {CO}_2$ uptake from Silicate and Carbonate Weathering; ICW Inorganic Carbon input from Weathering; Bu: Burial; PF: Photosynthetic fixation; RZ: Riparian Zone. Meaning, definitions, source literature of fluxes F1–F4 and of all other arrows are reported in Supplementary Table S1. (**b**) In meandering rivers, channel-migration-driven capture of woody biomass is exported from the outer bank into the stream (eCE). Young biomass then colonizes the inner newly deposited point bar, driving further $\hbox {CO}_2$-fixation from the atmosphere (Enhanced Net Primary Production - ENPP), stabilizing the bar and promoting further river migration (feedback effect). Hydraulic energy (dashed blue arrows) drives morphodynamics and channel migration, while solar energy (dashed yellow arrows) drives the consequent $\hbox {CO}_2$-fixation from the atmosphere. The output of the pump is the mobilization of LWD and POM, which is eventually stored in river channel sediments downstream (sediment spiralling) or farthest in oceans.

Like the biological carbon pump^14^, whereby phytoplankton net production and its ultimate marine fall drive carbon from the atmosphere to ocean interior and seafloor sediments, we conjecture that photosynthetic fixation by riparian vegetation, the recruitment of riparian vegetation, its transport, and burial, fit together in an integrated nexus in which rivers drive a carbon pump from the atmosphere to long-term stocks (i.e. floodplains and ocean). We conjecture that carbon mobilization is triggered by a two-step pumping mechanism. The first step refers to the eco-morphodynamic Carbon Export from floodplains (synthetically referred to in the following as eCE), whereas the second step, namely the Enhanced Net Primary Production (ENPP), consists of C-fixation promoted by vegetation encroachment on bare riparian areas generated by the morphodynamic activity. We, therefore, define the eco-morphodynamic Carbon Pump (eCP) as the combination of these two processes, that work in cascade, and that are mainly energized by channel migration in meandering rivers (Fig. 1b) and by overflow and flooding in multi-thread rivers. The former are single channels with a sinuous planform comprising a series of regular curves (meanders) moving and evolving in time. Meander migration is due to bank erosion on the outside bank of curved channels and point bar and floodplain generation on the inside bank. The latter are characterized by the occurrence of several interconnected channels separated by mid-channel bars or islands encroached by vegetation.

River systems store organic carbon in four interconnected compartments^15^: (a) Standing riparian biomass; (b) Large downed wood (> 10 cm in diameter and 1 m in length); (c) Sediments, litter humus, and soil organic carbon (SOC); (d) In-stream biomass which decomposition process produces Particulate Organic Matter (POM) and Dissolved Organic Matter (DOM). In this paper, we refer to carbon fluxes of live woody vegetation as the wood directly recruited from compartment (a) and delivered to the other compartments through bank erosion, flooding, uprooting and burial. We do not focus on SOC, whose dynamics have already been well explored elsewhere^16,17^.

Notably, a quantification gap can be highlighted in the latest calculations of the carbon cycle budget, whereby the LWD component of the aquatic-floodplain-estuarine flux remained unexplored^4–7^. In fact, the global estimates (Fig. 1, Supplementary Table S1) of about 2.9–8.3 PgC/year of the carbon terrestrial export from fluvial sediments and riparian vegetation to inland waters were obtained by subtracting the out-fluxes—i.e., out-gassing (2.1–3.9 PgC/year, Ref.^7,18^), burial (0.6–4.2 PgC/year, Ref.^7,17^), and the oceanic export ( 1 PgC/year, Ref.^19^)—from the in-fluxes, i.e., bed-rock weathering (0.5 PgC/year, Ref.^20^) and in-stream autochthonous photosynthetic fixation (  20% of the out-fluxes, Ref.^19^). However, this budgeting overlooks LWD recruitment in the export, since it is reasonable not assuming that the whole woody input is decomposed and reduced to micrometric size (traditionally considered < 20 $\upmu$m) during the transit time in the fluvial system, and hence not stating that transported wood is transformed into the fine fraction of POM or it is mineralized. Furthermore, recent assessments of global $\hbox {CO}_2$-evasion rates^18,21^ and inland water surfaces^22^ do not consider in the budget the vegetation recruitment to flow downstream, transport, deposition, and burial in the floodplain^6,10^. Since the main source of LWD arises from plant uprooting due to overflow and bank erosion, logs tend to be routed during floods, and floating logs can be buried in the stream and alluvial sediment surviving for millennia before decomposing^11^. The organic carbon in the form of POM or LWD can be deposited under anoxic conditions and long-term stabilized^23,24^. In addition, the high migration rates in lowland meandering rivers (e.g., 0.03–0.05 channel width per year^25^) may reduce the residence time of floodplain-stored material^13^, and therefore limit the time available for oxidation^26^. Although a fraction of the carbon recruited by the river returns to the atmosphere through processes of decomposition^27^ and out-gassing^28^, there is a part that is permanently stocked within river corridors as sedimentary organic carbon^13^, or delivered to coastal zones and deep oceans^19^. Due to these processes, large lowland floodplain systems have been in fact recognized to be significant carbon sinks^29^. On the other hand, LWD does not necessarily remain on a consistent downstream journey in the river and may spend significant time in logjams or deposited onto the floodplain^30^. A quantification of the role of LWD in the carbon budget of the aquatic-terrestrial transitional zone seems to be needed^31^ and a knowledge gap and the uncertainties in the fluxes reported in Fig. 1 were already remarked^7^. Three aspects deserve further investigation: (1) The recruitment of LWD flux seems to be generally underestimated at the global scale, although it is strongly associated with river morphodynamic processes nevertheless; (2) Periodic rejuvenation of the riparian vegetation triggers a yet unexplored enhancement of the net primary production of the fluvial corridor, with a direct effect on the carbon budgeting; (3) The fate of LWD fluxes after the recruitment from the riparian zone still lacks a quantitative global estimation^6^, in particular concerning the effect of burial in tropical floodplains^10,15^, in lakes and estuaries^32^. By conjecturing and quantifying the Eco-morphodynamic Carbon Pump (eCP), the present work quantitatively addresses point (1) and discusses the effects on point (2) for the Neotropical region (namely the most C-active in the world).

The first step of the pumping mechanism comprises the stream-induced biomass recruitment of LWD from standing riparian vegetation by erosion and flood-induced uprooting. This biomass is uprooted and/or transported into the water stream (or it remains downed in situ) and either stocked somewhere through burial in the fluvial floodplains or delivered to the oceans. The second step ENPP is C-fixation promoted by vegetation encroachment and primary production on new bare riparian areas. These two steps involve two mutually compensating carbon fluxes crossing the riparian zone, respectively outgoing and incoming.

## Results

### Study design

We analyzed the dynamics of tropical rivers in the Neotropics wider than 200 m in the period 2000–2019 and previously classified as free-flowing^33^, i.e., weakly disturbed by anthropic activities. This resulted in a dataset of 80 large rivers embedded in 235 regions of interest (ROI), with a total fluvial length of 59,000 km and a total analyzed floodplain area of 302,000 $\hbox {km}^2$ (i.e., one-sixth of the global extent of floodplains, according to Ref.^34^). Through remote sensing analysis of satellite datasets developed on the cloud computing platform Google Earth Engine (Supplementary Table S2) we focused on the area in the river corridor that had a vegetation loss due to river dynamics. With a probabilistic classification mapping (“Methods”), starting from a 30m resolution Landsat-based product^35^, this area was estimated to be 12,125 ± 286 $\hbox {km}^2$ in the period 2000–2019, which corresponds to an average annual forest loss of 638 ± 15 $\hbox {km}^2$/year.

### Carbon export in the Neotropics

**Figure 2:**
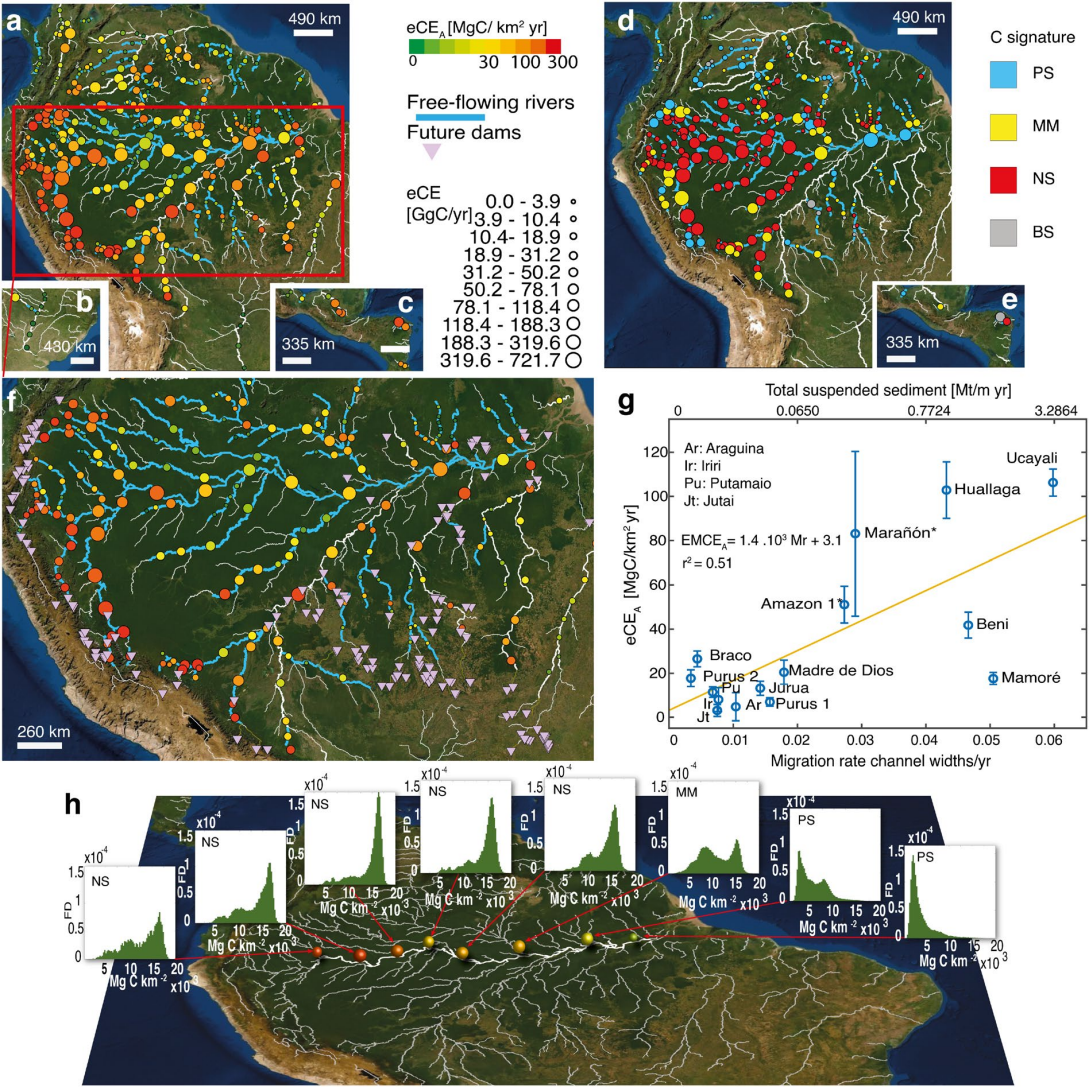
Eco-morphodynamic Carbon Export (eCE) and carbon signature of the largest rivers in Tropical America. The eCE in (**a**) South America, (**b**) Paraguay-South Brasil, (**c**) Central America. Carbon signature in (**d**) South America, (**e**) Central America. Point size is proportional to eCE, colors show eCEA (**a**–**c**, **f**, **h**) and signature (**d**, **e**). Blue reaches indicate free-flowing streams (CSI index > 95%, after Ref.^33^). See Supplementary Discussion for details about the analysis of an additional group of rivers (defined as moderately altered by Ref.^33^, not considered in the main analysis). (**f**) Magnified view of Andean-foreland forest basin and distribution of planned new large hydroelectric dams (>1 MW, see Ref.^36^) shown by pink triangles. (**l**) Correlation between sediment transport, migration rate, and carbon export (data on migration rate and sediment transport from ref.^25^, in the river marked with * the migration rate was derived from the relationship Mr = 0.043.$\hbox {TSS}^{0.28}$, as suggested by Ref.^25^, where TSS is the total suspended sediment. (**h**) The longitudinal sequence of signatures in the frequency distribution (FD) for Amazon River corridor biomass density (NS, negatively skewed; MM, multimodal; PS, positively skewed.)

Forest losses were combined with biomass densities to assess the strength of the Eco-morphodynamic Carbon Export (eCE) and its value per unit ROI area: eCEA (“Methods”). We estimated that large tropical rivers in the Neotropics export 8.90 ± 0.84 TgC/year of woody biomass carbon from riparian corridors (eCEA= 29.5 ± 0.36 MgC/$\hbox {km}^2$year, Fig. 2, Table 1). Overall, 57$\%$ of the total carbon export is due to just five rivers (6$\%$)—Big exporters—each contributing eCE > 0.3 TgC/year. The areas dynamically affected by these rivers occupy 35$\%$ of the total area considered. They include: (1) Extensive Exporters (eCEA < 50 MgC/ $\hbox {km}^2$ year), which are major contributors due to their large fluvial corridors, such as the Rio Negro; (2) Intensive Exporters (eCEA > 90 MgC/ $\hbox {km}^2$ year) with less extensive fluvial corridors but high migration rates (Mr > 4 $\times$ 10$^{-2}$ channel widths per year, Ref.^25^) such as the Ucayali River.

**Table 1:** Estimates of Eco-morphodynamic Carbon Export (eCE) and River-Driven Forest Loss Area ($\hbox {A}_{\text{ RDFL }}$) for the largest tropical rivers.

Basins	$\hbox {A}_{ROI}$	$\hbox {A}_{\text{ RDFL }}$	eCE $\hbox {A}_{\text{ RDFL }}$
	[10$^3$ $\hbox {km}^2$]	[$\hbox {km}^2$/year]	[TgC/year]
America			
Upstream Amazon	75.2	295 ± 7.36 (± 2.5 %)	4.28 ± 0.1 (± 2.3 %)
Central Amazon	160.2	212 ± 10.83 (± 5.1 %)	3.13 ± 0.09 (± 2.9 %)
Downstream Amazon	35.6	94 ± 5.75 (± 6.1 %)	1.08 ± 0.05 (± 4.6 %)
Others	31.0	37 ± 4.67 (± 12.6 %)	0.41 ± 0.03 (± 7.3 %)
Big Exporters (eCE > 0.3 TgC/year)			
River	eCE [TgC/year]	River	eCE [TgC/year]
Amazon	2.6 [29.2%]	Purus	0.38 [4.3%]
Ucayali	1.4 [15.7%]	Maranon	0.31 [3.5%]
Rio Negro	0.42 [4.7%]		

Values in parentheses indicate the percentage error while values in square parentheses are the percentage relative to total eCE = 8.9 Tg C/year. Uncertainty analysis is described in “Methods”.

As pointed out by Ref.^25^, the whitewater rivers in the Andean-foreland basin—Ucayali, Huallaga, Beni and Maranon—are highly dynamic due to the high suspended sediment load they carry (0.23 ± 0.16 Mt/year). In fact, the suspended load in these catchments was found to be positively correlated with river migration rates^37^ because sediment transport increases the buildup of fluvial bars, which enhances the topographic steering of longitudinal flow^38^ and thus promotes shear stress and bank erosion^39^. Such a key phenomenon, combined with the nutrient-rich sediment and high biomass density (8–16 GgC/$\hbox {km}^2$) of fluvial corridors in the Andean-foreland basin, makes it the most active basin in the Neotropics for carbon transport (48.1$\%$ of the total eCE of large tropical rivers in the Neotropics). Our reanalysis of 14 selected rivers of the Amazon basin, whose migration rate per unit width Mr was already known, further suggests that eCE is positively correlated with Mr and/or the total suspended sediment TSS (Fig. 2g) and supports our hypothesis of morphodynamically driven carbon export.

### Catchment scale analysis of carbon export

The Amazon basin and the corresponding tributaries can be divided into three geomorphologically homogeneous sub-regions (Supplementary Fig. S1). The upstream region, corresponding to the Peruvian-Bolivian Amazon basin, is the most dynamic (eCE = 4.3 TgC/year) with high levels of sinuosity, bank erosion rate and channel migration. The lowland rainforests in such a region are heavily influenced by lateral erosion of meandering rivers and new sequential succession forest develops on scroll bars very rapidly, while most of the (mature) mosaic vegetation loss is on the outer bank or in the short-lived islands^40^.

The middle region (eCE = 3.1 TgC/year) is characterized by a lower erosion rate and more stable channel banks. Meandering rivers (e.g., such as Purus, Jurua, Jutai) have migration rates lower than 0.2 channel-widths/year^25^, due to the low levels of sediment transport (Fig. 2g) and $\hbox {eCE}_A$ = 1.4–100 MgC/$\hbox {km}^2$/year. The Amazon River corridor of this region is characterized by an increase in the recurrence of low-waters, with green grass and shrubs species colonizing a rising portion of wetlands with the consequent reduction of woody plant communities^41^. For instance, the Negro River corridor (a tributary of the Amazon River in Central Amazonia) is characterized by relatively lower biomass density where swamp forest (Igàpo) and white sand vegetation populate stable islands^42^.

The downstream subregion (e.g., Jurunea, Rio Mapuera) provides the lowest levels of carbon export in the Amazon basin (eCE=1.1 TgC/year, $\hbox {eCE}_A$ = 19.4 MgC/$\hbox {km}^2$ year). The Amazon River corridor is here populated by dish-shape lakes in the floodplain and herbaceous vegetation is widespread. Carbon recruitment is dominated by recurrent floods, so vegetation remains at an immature stage and biomass density is usually low (< 5.3 GgC/$\hbox {km}^2$). However, the amount of carbon exported remains high due to high river-land connectivity (0.8 TgC/year, in the Amazon river corridor alone) while eCEA is lower than the upstream zone. Other rivers outside the Amazon Basin (Orinoco basin) and the rivers of Central America sequestrate 0.41 TgC/year with $\hbox {eCE}_A$ that ranges between 1 and 102 MgC/$\hbox {km}^2$ year (mean value: 17 MgC/$\hbox {km}^2$ year).

### Carbon signature

From a planimetric point view, large unconfined fluvial systems that are characterized by river dynamics can be broadly divided into two groups: (1) multi-thread; (2) single-thread systems^43^. The former group refers to braided and wandering rivers that are elevation-dominated, whereby flooding removes or buries vegetation through large elevation change rates related to deposition/erosion. This maintains the fluvial system in a juvenile, but highly productive stage, in accordance with the Intermediate Disturbance Hypothesis^44^ and the Flood Pulse Concept^27^. The latter, single-thread systems, refer to sinuous/meandering streams that are planimetry-dominated, whereby lateral erosion and deposition act antithetically, thus producing vegetation reallocation in the fluvial corridor.

We observed that the eco-morphodynamic Carbon Export leaves a morphology-dependent footprint in biomass distribution, because of the downstream gradients in the waterlogging duration (also called hydroperiod) and fluvial planforms. Through the analysis of the WHRC Carbon Stock dataset^45^, and the use of a new clustering algorithm (“Methods”, Supplementary Fig. S2, Supplementary Table S3), we identified four signatures of fluvial biomorphological activity evident in biomass distributions within ROIs (Fig. 2d, e, h, Supplementary Table S3): negatively-skewed (NS, 47.9$\%$ of observations), positively-skewed (PS, 29.9$\%$), multimodal (MM, 16.2$\%$), bell-shaped (BS, 6.0$\%$). We observed that fluvial corridors follow the NS-MM-PS longitudinal sequence fairly closely with increasing the Horton-Strahler number^46^, a scenario that is evident in the Amazon River (Fig. 2h). Such signatures are a proxy for the export capacity of rivers and demonstrate the link between sediment transport, flood pulses, river morphodynamics, and carbon pumping.

In single-thread sinuous/meandering rivers with high migration rates, lateral erosion removes the mature forest, while sediment deposition provides new fertile ground for juvenile vegetation colonization. The hydroperiod (i.e., the mean duration of seasonal floods) is short enough to allow the forest to reach the mature condition and store a high amount of carbon. Instead, the point bars and bare banks are rapidly vegetated by seedlings and young trees with high sequestration capability but low carbon density. Thus, the carbon distribution is negatively-skewed with a peak representing the mature forest and a left tail due to seedlings (Supplementary Fig. S8a). In multi-thread (braided/wandering) rivers, intermediate-to-high fluvial disturbances affect the vegetation that populates islands and banks. At weakly disturbed conditions (short hydro-period), a mature forest populates islands, or central bars, in the inner cores while young trees develop along the banks. Such a mixture of mature and young vegetation is recognizable as a multi-modal distribution in carbon density (Supplementary Fig. S8b). With the increase of the Horton-Strahler order, the hydro-period typically increases, the development of mature vegetation in the island cores is progressively inhibited and the system remains at a juvenile stage, so inducing a positively-skewed carbon density distribution (Fig. 2h, Supplementary Fig. S8c).

## Discussion and conclusions

Similar to the C-sink triggered by erosion of topsoil layers^47^, we here claim that river morphodynamics induces the recruitment of Large Woody Debris (LWD) from riparian vegetation through erosion and uprooting and promotes colonization and rejuvenation of the riparian zone^48^. The more the carbon export induced by river dynamics, the larger area is freed, and the higher the colonization of new vegetation, thus fostering further NPP. If for any reason, lateral erosion, uprooting, and overflow are interrupted, then C-export would be reduced, and rejuvenation of the floodplain inhibited. The enhancement of the net primary production is a direct effect of the carbon export (Fig. 1b), and it represents a valuable ecosystem service of the Aquatic-Terrestrial Transitional Zone. Unlike the eCE, a direct assessment of the ENPP at the continental scale is awkward through remote sensing techniques. However, in the long term (roughly longer than the time required by vegetation successional pattern to reach the state of mature forest), and under stationary hydro-morphological conditions, neither vegetation biomass removal nor production prevails and eCE and ENPP should equalize each other. In fact, from a first-order analysis of carbon balance in lowland floodplains, Ref.^12^ demonstrated that lateral carbon fluxes (erosion and deposition) have the same order of magnitude of vertical carbon fluxes (primary production and respiration).

A similar concept of enhanced-NPP was proposed for headwaters catchments of the Southern Alps in New Zealand, through a 70 m resolution analysis, where high frequent landslide in steep lands mobilize soil and above-ground biomass^49^. In this way, an unusual proportion of vegetation was shown to be in an early successional stage, with NPP higher than mature forest^12^. We have instead considered the triggering of ENPP in the context of lowland tropical floodplains at a continental scale and, by focusing on the nexus between ENPP and river eco-morphodynamics, we have emphasized a carbon pumping mechanism (eCP) that remained unexplored so far. For instance, this nexus is evident in the Amazon basin, that, during the observation period (19 years) has lost 11,420 $\hbox {km}^2$ of vegetated area for erosion and uprooting, with an overall carbon export of 161 TgC. When referring to the estimates provided by Ref.^50^, this quantity is 25 times larger than the net primary production that a tierra-ferma mature forest would have produced over the same area, and in the same period, in the case of no bank erosion or uprooting. Since it is well-known that the total floodplain biomass and forest cover remains roughly constant at the multi-decadal time scale in unaltered or unregulated rivers^51^, this excess must be compensated with a NPP of riparian forest dramatically higher than tierra-ferma mature forest^51^. Notice that this key aspect is usually overlooked in the literature since proof of such a balance from NPP direct measurements would require a period of observation typically larger than the satellite image availability. This explains why a recent attempt in estimating carbon accumulation in the Ucayali River has been misinterpreted as a strong and unphysical imbalance between accumulation and export^52^.

In terms of areal efficiency, the eco-morphodynamic Carbon Pump of lowland tropical rivers is a high-performance machine. In the Amazon basin, the carbon exported annually per unit area of river-driven forest loss may be computed as eCE/$\hbox {A}_{RDFL}$ = 218–275 MgC/$\hbox {km}^2$ year (Table 1). This value is higher than other widely known fluxes of the carbon cycle, such as POC fluxes from eroded peatlands (< 78 MgC/$\hbox {km}^2$ year, Ref.^53^), the rate of carbon storage in upland blanket peatland (55 MgC/$\hbox {km}^2$ year, ref.^54^) and mass wasting in tropical steep lands (3–39 MgC/$\hbox {km}^2$ year, Ref.^55^). Furthermore, by examining the mineral weathering of silicate soils, we may refer to angiosperm-deciduous systems, which induce an estimated average loss rate of calcium ions of 4 Mg/$\hbox {km}^2$ year^56^. This corresponds to 2.4 MgC/$\hbox {km}^2$ year for the Urey reaction stoichiometry, a value 100 times smaller than the present process. Net oceanic upwelling C-flux per unit area due to thermohaline and Ekman circulations is instead a thousand times smaller^57^.

As suggested by Ref.^12^, coarse material is typically not sampled by standard POC collection approaches. It is therefore reasonable that a consistent fraction of the 8 TgC/year of eCE herein assessed for the Amazon basin must be added to the current estimate of organic carbon flux from the Amazon River to the ocean (about 31 TgC/year from Ref.^58^).

It is evident that eco-morphodynamic carbon pumping is a process closely linked to the ability of river systems to recruit vegetation and sustain the rejuvenation of the riparian corridor. However, the river activity—carbon export nexus is broken when fluvial connectivity is undermined by anthropogenic activities. For example, dams and reservoirs impact the frequency and duration of flood pulses in the river network^59^ and can reduce the input of bedload and suspended sediment to the downstream reaches^60^, resulting in channel narrowing and incision^61^. Lower flood pulses and sediment supply can also greatly alter riparian vegetation dynamics^62^ by reducing seedling establishment, increasing vegetation encroachment, and leading to even-aged riparian forests^63,64^. Furthermore, greenhouse gas (GHG) emissions from the decomposition of organic matter transferred or produced within the reservoir as aquatic biomass, can mine the so-called Carbon Intensity of dams, namely the $\hbox {CO}_2$-equivalent emissions per unit of electricity generated. Consequently, 10$\%$ of the world’s existing hydropower plants emit as great a quantity of GHGs as would equivalent fossil-fuel power plants^65^.

From the above considerations, it follows that the current policies on environmental flows (e-flow) assessment for the Neotropics require modification because they are merely based on water flow requirements in downstream reaches^59^. Strategic planning implies the enforcement of operational rules aimed at lessening the overall effect on hydrological and geomorphological processes, including dynamic flow releases^66^, flood pulses, and sediment dynamics, when specifying e-flows^67^. Pure hydrology-based methodologies for e-flows assessment^68^ incompletely capture changes in channel morphology and vegetation dynamics.

Our analysis suggests that such actions should be recommended in dam design, involving at least the headwaters of the big sequestrators, which account for 28.2$\%$ of the total eCE (excluding the Amazon River, Table 1). A carbon budget approach to managing regulated rivers in the Neotropics is therefore essential to determine whether hydropower can be considered a clean energy source in the future.

The present result shows that neglecting the eCE could underestimate the current aquatic-terrestrial carbon flux by up to 8.9 TgC/year in the large rivers of the Neotropics alone (about 23% of current estimates of organic carbon flux to the ocean from major rivers in the Neotropics^58^). Furthermore, the result of the eCE in Neotropics has been conservatively underestimated. We in fact focused the main considerations on Neotropics watercourses with channel widths greater than 200 m, due to the resolution of the datasets adopted. The analysis could be globally extended to all natural large rivers.

Instead, nothing can be said for rivers smaller than 200 m, but it is worth remarking that they globally represent about 99.5$\%$ (in length) of the waterways^69^. In addition, headwaters are recognized as conveyors of coarse woody material and producers of POC^12^. Not considering them in a global estimate is therefore a further source of underestimation. Finally, to get a more comprehensive evaluation, in the spirit of the REgional Carbon Cycle Assessment Project (RECCAP2) initiative of the Global Carbon Project, and since the procedure is based on a freely available satellite imaging algorithm that does not require any calibration, it is recommended to extend the present assessment to non-tropical regions.

## Methods

### Definition of the regions of interests (ROIs)

Each analyzed river was divided into Regions of Interest (ROIs) characterized by homogeneous morphological behaviour. The ROI represents the elementary unit for the calculation of eCE and is characterized by longitudinal and lateral boundaries. The changes in Horton-Strahler order^46^, sinuosity, transitions from single-thread to multi-thread or vice versa, or sudden changes in channel width^22^ were considered as geomorphological criteria longitudinal divides between two consecutive ROIs (*sensu* Ref.^70^). The main channel of the analyzed rivers has a width greater than 200m, when referring to the mean annual discharge, according to the analysis by^22^. The lateral extent of ROIs comprises the land adjacent to the stream where vegetation is influenced by river dynamics and/or flooding. Such an active lateral area was identified in two steps.

First, it was considered the spatial gradient in biomass density. The areas frequently involved by flooding or river dynamics are featured by vegetation adapted to survive and are characterized by a successional pattern with specific biomass distributions^51^. An analysis of a high-resolution biomass map allowed us to identify edges between floodplain forest and land forest (e.g., defined as tierra-ferma in Amazonian basins, Supplementary Fig. S3)

Second, where the lateral boundaries were not evident by biomass map, we also considered the water surface occurrence by using the GSW dataset^71^, n.4 in Supplementary Table S2. Accordingly, the identification of sites ever detected as water over the last 35 years in the GSW dataset enabled us to identify the aquatic-terrestrial transitional zone.

Nevertheless, short-lived events are not always correctly detected by GSW because such events must be concurrent with cloud-free satellite observations. Because of the extreme cloud contamination that characterizes the tropical area (particularly the eastern Amazon Basin), many short but intense events cannot be included in the event map developed by^71^, making our estimates of the lateral boundaries of the ROIs further conservative.

### Data filtering

To ensure the quantification of carbon exported that was strictly based on River-Driven Forest Loss (RDFL), a three-step selection procedure was used to identify and exclude non-RDFL cases, e.g., rivers impacted by anthropic activities.

*Step 1*: All evident sources of anthropic alteration were identified by visual inspection from Landsat images, such as physical infrastructures in the river channel or along the surrounding floodplain, presence of river channelization, check dams, weirs, fords, embankments, bank protection, revetments, and mining activities.

*Step 2*: Rivers classified as not free flowing through the CSI index by^33^—i.e., rivers in which fragmentation and regulation or alteration in water quality and temperature compromise fluvial connectivity (CSI index < 95$\%$, dataset n.8 in Supplementary Table S2)—were also excluded.

These first two steps excluded 89$\%$ of 551,000 km overall length of all tropical reaches with Horton-Strahler index $\le$ 4.

*Step 3*: A probabilistic classification model was used to define the likelihood P that river-driven forest loss (RDFL) occurred for each pixel within the ROIs. Extreme likelihood values are P = 0 (no forest loss or forest loss unquestionably due to causes other than river dynamics), and P = 1 (forest loss unquestionably due to river geomorphic activity).

To assess intermediate probabilities, the Global Forest Change dataset^35^ was combined with three potential causes of non-river-driven forest loss: (1) population density; (2) forest fires; (3) land-cover changes (source datasets are described in Supplementary Table S2). For any pixel k of ROI j in which forest loss occurred, the model assessed the likelihood that the forest cover change was not due to urbanization ($\hbox {P}_{jk}^{(u)}$), wildfire ($\hbox {P}_{jk}^{(wf)}$) or man-made land-cover changes ($\hbox {P}_{jk}^{(lc)}$), thus yielding three probability maps (see next section). The overall likelihood map was obtained by multiplying the three probability maps, since they refer to independent events, namely $\hbox {P}_{jk} = \hbox {P}_{jk}^{(u)} \cdot \hbox {P}_{jk}^{(wf)} \cdot \hbox {P}_{jk}^{(lc)}$. In this way, an average annual area of 115 ± 15 $\hbox {km}^2$ in the tropical wetlands of large rivers was classified as non-RDFL and therefore excluded from the whole analysis. This corresponds to 18$\%$ of the annual cover loss detected in the ROIs. The results of the filtering procedure for three example cases are shown in Supplementary Fig. S5. The data used in this study refer to the HydroRIVERS data layers—n.9a in Supplementary Table S2^72^—providing vectorized line network of all global rivers with a catchment area greater than 10 $\hbox {km}^2$ or an average river flow larger than 0.1 $\hbox {m}^3$/s, and were derived from HydroSHEDS data—n.9b in Supplementary Table S2^73^—based on a grid resolution of 15 arc-seconds. River order was expressed using the Horton-Strahler ordering system. Following this system, the first order represents headwater streams and when two streams with the same order meet, they form a river of one order greater.

### Probabilistic classification model

A probabilistic classification model was used to define the likelihood (P) that a River-Driven Forest Loss (RDFL) had occurred for each pixel within the ROIs. To this aim, the Global Forest Change dataset^35^ was filtered by considering three potential causes of no river-driven forest losses: (1) population density; (2) forest fires; (3) land-cover changes (datasets n.5-7 of Supplementary Table S2). Accordingly, for any j-th pixel of the k-th ROI wherein forest loss occurred, the model assessed the likelihood that the forest cover change is not due to urbanization $\hbox {P}_{jk}^(u)$, wildfire $\hbox {P}_{jk}^{(wf)}$, or man-made land-cover changes $\hbox {P}_{jk}^{(lc)}$, thus producing three probability maps.

The values reported in the map $\hbox {P}_{jk}^{(u)}$, decrease with the population density (PD). According to a relationship between the human pressure score and the population density for sparsely populated areas suggested by Ref.^74^, we set:

1 $$\begin{aligned} P_{j,k}^{(\text {u})}={\left\{ \begin{array}{ll} 1-0.333\cdot \log {(\text{ PD }\!+\!1)}, &{}\quad \text{ for } \text{ PD }<1,000\quad \text{ people/km}^2\\ 0 &{}\quad \text{ for } \text{ PD }\ge 1,000\quad \text{ people/km}^2  \end{array}\right. } \end{aligned}$$

Human population density (PD) was retrieved from the dataset WorldPop Project Population (86–88, 91) at 100 m resolution (n.6 of Supplementary Table S2)

To define the maps $\hbox {P}_{jk}^{(wf)}$ and $\hbox {P}_{jk}^{(lc)}$, the probability that the forest loss in a given year has not been caused by a non-River-Driven Event (henceforth referred non-RDE) was expressed as a function f($\Delta$t), where $\Delta$t is the time gap (causal relation principle) between the forest loss and non-RDE occurred in the same region (wildfires or land cover changes). The function f($\Delta$t) (the probability that the loss has been caused by a non-RDE) follows a piecewise dependence on time, as reported in Supplementary Fig. S4.

Essentially, if the forest loss and the non-RDE belong to the same year (i.e., $\Delta$t =0), the causal connection is guaranteed, so the function f takes the maximum (f =1). Cases with $\Delta \hbox {t}<0$ mean that the non-RDE anticipated a forest loss. In this case, a positive causal connection may be possible for several reasons. For example: (1) the non-RDE might have not caused a detectable forest loss in the same year, e.g., a wildfire that irreversibly damaged the vegetation which however died in the following months/years; (2) extreme cloudiness of tropical region caused a delay in the forest loss detection. In the cases with $\Delta \hbox {t} >0$, forest loss anticipated the non-RDE. Albeit counter-intuitive, even in this case, a positive causal connection can be possible. For example, a slow land conversion (e.g., from forest to cropland) that takes some years to cover a portion of territory observable through a MODIS-based dataset (coarse resolution 500 m) while was suddenly detected as forest change in the Landsat-based products (resolution of 30 m). In each plot performing a forest loss during the observation window, fire events were detected by using the MODIS-based dataset^75^. We set

2 $$\begin{aligned} P_{j,k}^{(\text {wf})}=\prod _{i=1}^N 1-f_i(\Delta {t}), \end{aligned}$$

where N is the number of fires observed during 2000–2019 in the pixel. Where no fires were observed, $\hbox {P}_{jk}^{(wf)}$ =1. We remark that this filter excludes the capture of recalcitrant LWD generated by the incomplete combustion of biomass during fires, so-called black carbon as analyzed in^76^. This aspect may be an additional source of underestimation of the present eCE assessment.

The map $\hbox {P}_{jk}^{(lc)}$, namely the likelihood that forest loss is not due to land cover change caused by human activity, is generated by using the dataset n.7 in Supplementary Table S2, MODIS Land Cover Type MCD12Q1^77^. Following the classification of the Annual International Geosphere-Biosphere Programme (IGBP, Supplementary Table S4), four land cover macro-classes were identified: Natural with High vegetation density (NHV), Natural with Low vegetation density (NLV), Anthropic (AN) and Water/Unvegetated (UV). A per-pixel analysis at MODIS scale was performed in ROIs and each yearly variation in land cover macro class was detected and classified. In each pixel, the variations from NHV to NLV, from NHV to AN and from NLV to AN were considered due to human activities while all the other changes were attributed to river morphodynamic processes (i.e., RDFL). The probability that the forest loss at pixel k of ROI j was not due to human–induced land cover change is therefore defined according to the same equation as Eq. (2) where N is intended as the number of land cover transitions observed during the 2000–2019 in the same pixel, while $\Delta$t is intended as the time difference between the forest loss and the land cover change. When no human-induced land cover variations were detected, $\hbox {P}_{jk}^{(lc)}$=1.

For the above reasons, a conservative choice in terms of eCE estimation was to assume that when forest loss and non-RDE occurred within the temporal window of 5 years they were causally connected, so f =1. The result of the filtering procedure for three example cases is shown in Supplementary Fig. S5.

### Computation of the eco-morphodynamic carbon export (eCE)

The eCE of j-th ROI, reported as the TgC exported per year (in the form of woody biomass), was computed as

3 $$\begin{aligned} eCE_j=\sum _k eCE_{j,k}= \sum _k L_{j,k}\cdot \rho _{j,k} \end{aligned}$$

where $\rho _{j,k}$ is the biomass density [TgC/$\hbox {km}^2$] and $\hbox {L}_{j,k}$ is the annual mean RDFL [$\hbox {km}^2$/year] for the period 2000–2019, and for pixel k of ROI j. In order to statistically exclude non-fluvial causes, $\hbox {L}_{j,k}$ was computed as the product between the surface $\hbox {A}_{j,k}$ of the cell and the likelihood $\hbox {P}_{j,k}$ of loss being RDFL (see the section “Probabilistic Classification Model”). For the assessment of biomass density, we adopted four different methods (M1–M4).

*Method M1*: $\rho _{j,k}$ was taken from the WHRC Carbon Stock dataset developed by^78^ for the above-ground living woody biomass density at 30 m resolution for the year 2000 (n.2 of Supplementary Table S2). In this case, the carbon density of a single cell was assumed constant during the entire period of analysis, neglecting the possibility that plots, where the loss occurred after the year 2000, might have experienced an increase in the carbon content due to growth in the time between 2000 and the year of loss.

*Method M2*: The value of carbon density of each pixel was adjusted considering the amount of vegetation that had grown between the year 2000 and the year of loss, by using a calibrated logistic growth model (see next section).

*Methods M3 and M4*: The value of carbon density of each pixel was approximated using the spatial average over the whole ROI (i.e., $\rho _{j,k} = \sum _k \rho _{j,k}/N_j$, being $N_j$ the number of pixels in ROI j) by using the WHRC *Carbon Stock* datasets by^78^ for M3 and^45^ for M4 (datasets n.2 and n.1 of Supplementary Table S2, respectively). These datasets describe biomass in tropical regions for only a limited period (the year 2000 for n.2 and the period 2007–2008 for n.1).

Tropical rivers are highly dynamic systems that during an inter-decade evolution likely visit most of their geomorphological configurations (e.g., the Ucayali River, a tributary of the Amazon River, shows migration rates of up to 100 m/year). For methods M3 and M4, we, therefore, adopted an ergodic-like hypothesis^79^, which allowed the temporal mean of carbon density in a single plot to be inferred from its spatial average over the whole ROI. It is worth noting that spatial averaging in methods M3 and M4 induces a slight underestimation of the eCE (see Supplementary Table S5), since the erosion mechanism and the consequent capture of biomass usually involve the mature bank, where vegetation is at a higher level of growth.

Since the considered datasets only report the above-ground biomass (AGB) density, the belowground biomass (BGB) was assessed as BGB = 0.489 $\cdot$ $\hbox {AGB}^{0.89}$ (Ref.^80^), and the total carbon was estimated as 50$\%$ of the total biomass (AGB+BGB). From the estimates of eCE, we also estimated the carbon sequestrated per unit ROI area and per river length:

4 $$\begin{aligned} eCE_A= {} eCE/S_r\ \ \text {[kg C/}\hbox {m}^2 year] , \end{aligned}$$

5 $$\begin{aligned} eCE_L= {} eCE/l\ \ \text { [kg C/m year]}, \end{aligned}$$

where $S_r$ is the ROI surface, and $l_r$ is the length of river reaches within the ROI. We remark that the relative differences of the eCE estimation among methods M1-M4 does not exceed 3.3$\%$ (Supplementary Table S5). Quantitatively, the four different methods, therefore, perform in a very similar way, despite they are based on different datasets. For simplicity, the results reported in the main text refer to Method 2. A graphical summary of the whole methodology is reported in Supplementary Fig. S6.

### Calibration of the logistic growth model update in Method M2

In method M2, the increase in the carbon content, due to vegetation growth between the acquisition time (year 2000, ref.^78^) and the time of forest loss, was considered by calibrating a simplified logistic biomass growth model^63,81^,

6 $$\begin{aligned} \frac{d\rho _i}{dt} = \alpha _i \rho _i\left( V_i-\rho _i\right) \end{aligned}$$

where $\rho$ is the biomass carbon density, *t* is time, *V* is the carrying capacity, i.e., the maximum sustainable biomass carbon density, and $\alpha$ is the species-dependent growth rate, while subscript *i* refers to the generic *i*-th cell. By setting the initial condition $\rho _{0,i}$ = $\rho (t_0)$, that corresponds to the biomass reported by the dataset^78^ at year $t_0$ = 2000, the formal solution of equation (6) at time $t=t_0 +\Delta t$, for a generic species community reads

7 $$\begin{aligned} \rho _i = \frac{A_i \rho _{0,i} V_i}{\left( A_i-1\right) \rho _{0,i}+V_i} \end{aligned}$$

where we have defined $A_i=\hbox {e}^{V_i \alpha _i \Delta t}$. Through the following procedure, we have locally calibrated the function $\hbox {A}_i$ and the parameter $\hbox {V}_i$, in order to use the Eq. (7) to update the value of carbon biomass density from $t=t_0$ = 2000 to the time of the cover loss ($t=t_0+\Delta t$), in any cell. The calibration procedure relies on the comparison of carbon biomass as reported by two different datasets with acquisition times eight years apart (Ref.^78^ and Ref.^45^ referring to 2000 and 2008, respectively n.2 and n.1 in Supplementary Table S2). The comparison of these two datasets is possible since they were generated by the same methodology, albeit with different resolutions (30 mpx for Ref.^78^ and 500 mpx for Ref.^45^). In the following, the two datasets will be tagged with subscripts $_{30}$ and $_{500}$, respectively. Firstly, all cells in the 30 m resolution dataset were resampled to the 500 m resolution within blocks corresponding to the pixel boundaries of the second dataset. Secondly, for each j-th block, we imposed the matching between the mean of the values $\rho _{30,i}$ within the block (updated at t = 2008) and the value $\rho _{30,i}$ , namely,

8 $$\begin{aligned} \frac{1}{N_j}\sum _{i=1}^{N_j}\left[ \rho _{30,i}\right] _{t=2008}= \rho _{500,j} \end{aligned}$$

which, after using Eq. (7), becomes

9 $$\begin{aligned} \frac{1}{N_j}\sum _{i=1}^{N_j}\frac{A_i \rho _{0,i} V_i}{\left( A_i-1\right) \rho _{0,i}+ V_i} \vert _{\Delta t= 8\ \text {years}} = \rho _{500,j}, \end{aligned}$$

where $\hbox {N}_j$ is the number of 30 m resolution cells in the j-th 500 m resolution block. Third, it was assumed that all cells within each block share the same value of $\hbox {A}_i$ and V, so $\hbox {A}_i$ = A is a constant which can be taken out from the summation in Eq. (9). Furthermore, since 1/$\rho _{0,i}$ (A–1) $\approx 1/ \rho _{0,i} \gg$ 1/V, as a first order approximation we get

10 $$\begin{aligned} A \vert _{\Delta t^*} \approx \frac{\rho _{500,j}}{\rho _M}, \end{aligned}$$

where $\rho _M$ = $N_j^{-1} \sum _{i=1}^{Nj}\rho _{0,i}$. By iterating and substituting Eq. (10) in Eq. (9), one gets a second-order approximation

11 $$\begin{aligned} A \vert _{\Delta ^*} \approx \frac{N_j \rho _{500,j}}{\sum _1^{N_j} \frac{1}{\frac{\frac{\rho _{500,j}}{\rho _M}-1}{V}+\frac{1}{\rho _i}}} \end{aligned}$$

By recursion, it is evident that further approximations lead to a cumbersome formula containing a continued fraction in the denominator of Eq. (11), and for numerical convenience, it suffices to stop at the second step. The carrying capacity was cautiously assumed constant throughout the ROI and equal to the maximum value of $\rho _M$ (namely, V = $\rho _M^{max}$).

By replacing in Eq. (7), and after recalling that, by definition,

12 $$\begin{aligned} A \vert {\Delta t}= \left( A \vert _{\Delta t ^*}\right) ^{\frac{t}{\Delta t^*}} \end{aligned}$$

where $\Delta t^*$ = 8 year is the time lag between the two datasets, one finally gets the relationship for the carbon density updated at time t, for each cell:

13 $$\begin{aligned} \rho _i(t) = \frac{\left( A\vert _{\Delta t^*}\right) \rho _{0,i}\rho _M^{max}}{\left[ \left( A \vert _{\Delta t^*} \right) ^{\frac{t}{{\Delta t^*}}}-1\right] \rho _{0,i}+ \rho _M^{max}} \end{aligned}$$

An example of use of Eqs. (11)–(13) is reported in Supplementary Fig. S7.

### Identification of the biomass distribution signatures

The analysis was performed using a set of Java APIs (Application Programming Interface), that are optimized for the analysis of big data. Due to the lack of an efficient and simple procedure for multi-modality detection^82^, we also developed an ad-hoc signature classification algorithm that is able to distinguish four patterns in the biomass density distributions (negatively skewed, positively skewed, multi-modal, bell-shaped). The procedure combines the statistical parameters of the carbon density distribution across the ROI: mode (M), median (Med) and skewness (Sk). These parameters were calculated using a set of GEE-native geo-statistical functions, applied to the WHRC Carbon Stock Dataset^45^. The minimum bin of histograms was fixed to 200 MgC/$\hbox {km}^2$ (the accuracy reported by^45^ is 100 Mg C/ $\hbox {km}^2$).

Firstly, the algorithm (Supplementary Fig. S2) separates uni-modal from multi-modal distributions. To this aim, two sub-samples are extracted from the dataset of each ROI, by considering a cutoff at the median value of the carbon density distribution, referred to as the left—(L) and right—(R) sub-samples. ROIs’ distributions are classified as multi-modal (MM) if two conditions are both satisfied:

(i) The frequency of the mode of the left ($\hbox {F}_ML$) or right ($\hbox {F}_MR$) sub-samples exceeds more than ± 10$\%$ the frequency of median value of the whole sample ($\hbox {F}_{Med}$);
(ii) Left (ML) or right (MR) modes are distant enough to the main median (Med), namely: |$\hbox {M}_{L,R} - \hbox {Med}| > 400$ MgC/$\hbox {km}^2$.

If conditions (i) and (ii) are both false, the skewness Sk of the main distribution is considered: positive skewness (Sk > 0.4) provides PS, negatively skewed (Sk $> -$ 0.4) provides NS, whereas moderate skewness (− 0.4 < Sk < 0.4) provides BS distributions. If only one of either (i) or (ii) is satisfied (i.e., just one sub-sample mode is detected to be distant from the median) the difference D = $\hbox {F}_{MR} - \hbox {F}_{ML}$ is computed to distinguish between NS (D < 0) and PS (D > 0) distributions. If the condition related to D is not satisfied the algorithm uses again $\hbox {S}_k$ to classify biomass density distributions in NS, PS or BS classes.

The algorithm was tested on a subset of 10$\%$ of the ROIs, randomly selected as a possible validation dataset, which showed a total accuracy of 95$\%$ (correctly classified distributions). An example of application on three rivers is reported in Supplementary Fig. S8. The Matlab code is reported in the Online supplementary material (Figshare repository).

### Uncertainty analysis

The aggregated continental assessment of eCE for the largest tropical rivers was obtained as the sum of the values calculated in each ROI of the continent. The uncertainty (namely the standard deviation, henceforth referred to with symbol $\sigma$) at the pixel level may be computed from probability theory. The eCE is in fact the product of two quantities both affected by error (i.e., river-driven forest loss area and biomass carbon density) so they can be considered as random processes. According to the filtering procedure described above, for each pixel, forest loss can be associated with a discrete random variable $\chi _{j,k}$ that takes only two values: 1 with probability $\hbox {P}_{j,k}$ (RDFL) or 0 with probability 1–$\hbox {P}_{j,k}$ (non-RDFL). This corresponds to a Bernoulli process^83^—i.e., repeated coin flipping—, which has the mean equal to $\hbox {P}_{j,k}$ and variance equal to

14 $$\begin{aligned} \sigma ^2\left( \chi _{j,k}\right) = P_{j,k} \left( 1-P_{j,k}\right) . \end{aligned}$$

The carbon density is instead a continuous random variable, with mean $\rho _{j,k}$ (from methods M1–M4) and standard deviation $\sigma (\rho _{j,k}$). By using Goodman’s expression^84^ for the variance of a product of two uncorrelated random variables, the error variance of the eCE of pixel (j,k) reads

15 $$\begin{aligned} \sigma ^2\left( eCE_{j,k} \right) = A^2_{j,k} P_{j,k} \left[ \sigma ^2\left( \rho _{j,k}\right) +\left( 1-P_{j,k}\right) \rho _{j,k}^2\right] , \end{aligned}$$

where $\hbox {A}_{j,k}$ is the pixel area. Per-pixel values for $\sigma (\rho _{j,k})$ are not reported in the raw datasets herein considered, so we adopted different conservative assumptions, based on the observation that residuals are proportional to the mean, as also suggested in ref.^45^. Accordingly, in M1 and M2, we set

16 $$\begin{aligned} \sigma \left( \rho _{j,k}\right) = c_V \cdot \rho _{j,k}, \end{aligned}$$

with the coefficients of variation $\hbox {c}_V$ assuming different values ranging between 0.5 and 1.25.

In M3 and M4, $\sigma (\rho _{j,k})$ was set equal to a constant value throughout the ROI, corresponding to the standard deviation of all the carbon densities measured inside the ROI, as reported in the *WHRC Carbon Stock* datasets^78^ for M3, and in the dataset by Ref.^45^ for M4.

As a further step, the propagation of the uncertainty from the pixel to the continental scale requires the assessment of the spatial correlation of the errors, otherwise per-pixel errors cancel out and the overall uncertainty may be largely underestimated. In the present case, standard use of spatial variograms (*sensu* ref.^45^) is precluded by the spatial patchiness of ROIs, the heterogeneity of biomass due to river dynamics and in addition, because it is computationally prohibitive (even when encoded in GEE). Following Ref.^45^, we therefore adopted two empirical autocorrelation length-scales (ALS) - $\hbox {ALS}_1$ equal to 500 m and $\hbox {ALS}_2$ equal to the ROI area—and we conservatively assumed that the pixels are perfectly correlated at a distance smaller than the ALS and uncorrelated at larger distances. The dataset was divided into independent blocks by using squares (for $\hbox {ALS}_1$) or ROI polygons (for ALS2) and an upper conservative estimate of the uncertainties was calculated for each block by exploiting all the values of $\sigma$ ($\hbox {eCE}_{j,k}$) provided by aforementioned Goodman’s formula within the block. For each method M1–M4, the uncertainty in the eCE at the continental scale $\sigma _{cont,ALS}$ was calculated for both ALS, by summation of the variance associated with each block within the continent:

17 $$\begin{aligned} \sigma ^2_{cont,ALS}= \sum _{i=1}^N \sigma ^2_{i,ALS}, \end{aligned}$$

where N is the number of blocks in a continent and $\sigma ^2_{i,ALS}$ is the variance error associated with each block.

For methods M3 and M4, the errors were assessed only with $\hbox {ALS}_2$, since in both scenarios the carbon density was derived from a spatial average at the ROI scale. In each block, the variance was calculated by taking its supremum over the block, i.e.,

18 $$\begin{aligned} \sup _{(j,k)\in \text {block}}eCE_{j,k}. \end{aligned}$$

By combining methods M1–M4 with the two auto-correlation length scales and considering the four values of $\hbox {c}_v$ for methods M1 and M2, eighteen different configurations were considered for the uncertainty assessment (Supplementary Table S5). The most conservative configuration (maximum uncertainty) gives a standard deviation (in TgC/year) and a percentage error of 0.84 (9.44$\%$) for Neotropics. The results reported in the main text refer to this conservative configuration.

## Supplementary Information

Supplementary Information.

## Data Availability

The Java script for the GEE platform generating the row dataset, and the Matlab scripts generating the definitive data set are deposited in https://figshare.com/s/6b5347f0e1ad3d92a486, and freely available after publication.
