# Supplementary material for: Eco-morphodynamic carbon pumping by the largest rivers in the Neotropics
Source: Sci Rep. 2023 Apr 5;13:5591. doi: 10.1038/s41598-023-32511-w (PMC10076311; doi:10.1038/s41598-023-32511-w)
Supplement: Supplementary file 1 — Supplementary Information. [file 41598_2023_32511_MOESM1_ESM.pdf]

Supplementary Information for: Eco-morphodynamic carbon pumping by the largest rivers in the Neotropics

Luca Salerno[1,*], Paolo Vezza[1,+], Paolo Perona[2,+], and Carlo Camporeale[1]

[1]Department of Environment, Land and Infrastructure Engineering, Politecnico di Torino, Corso Duca degli Abruzzi, 10129, Torino, Italy
[2]Hydraulic Platform - LCH, Institute of Civil Engineering (IIC), School of Architecture, Civil and Environmental Engineering (ENAC), EPFL, CH
[*]luca.salerno@polito.it
[+]these authors contributed equally to this work

ABSTRACT

This PDF file include:

List of Abbreviations
Supplementary discussion
Supplementary Figs. S1-S8
Supplementary Table S1-S5
Supplementary References
Legend for datasets (spreadsheets) S6-S7

Other supplementary materials for this manuscript (reported in the figshare repository) include the following:

Supplementary datasets (spreadsheets) S6-S7
Matlab scripts
Google Earth Engine Scripts
User Guide with demo

List of Abbreviations

AGWB	Aboveground Biomass
API	Application Programming Interface
BS	Bell-Shaped
Bu	Burial
eCP	Eco-morphodynamic Carbon Pump
ENPP	Enhanced Net Primary Production
GEE	Google Earth Engine
GHG	Greenhouse gases
GPP	Gross Primary Production
GSW	Global Surface water
IGBP	Intern. Geosphere-Biosphere Progr.
ICW	Inorganic Carbon input from Weathering
IPCC	Intergovern. Panel on Climate Change
LWD	Large Woody Debris
MM	Multi-Modal pattern
MODIS	Moderate Resol. Imaging Spectroradiomete
M_r	Migration rate
NPP	Net Primary Production
NHV	Natural with High vegetation density
NLV	Natural with Low vegetation density
NPP	Net Primary Production
NS	Negatively Skewed pattern
PD	Population density
PF	Photosynthetic fixation
POM	Particulate Organic Matter
PS	Positively Skewed pattern
R	Respiration
ROI	Region Of Interest
RZ	Riparian Zone
SCW	atmospheric CO_2 uptake from Silicate and Carbonate Weathering
TSS	Total Suspended Sediment
WHRC	Woods Hole Research Center

Supplementary Discussion

In the context of water management policies, the issue of connectivity of river systems worldwide was recently assessed using an integrated index (sensu ref. [32]) that considers river fragmentation, flow regulation, sediment trapping, water consumption and infrastructure development. By using this index, we have found that an additional group of 88 river segments (not considered in the main analysis, Table S7), classified as moderately altered (i.e., CSI<95%, see white reaches in Fig. 2A-K), provide eCEA 21% lower than free-flowing rivers. This suggests that a loss of river connectivity reduces the Eco-Morphodynamic Carbon Export. This latter result reinforces concerns about the environmental vulnerability of rivers to dams in the Neotropics[86], where 101 large hydropower dams are planned in Peru and Bolivia with a total installed capacity of 46 GW[87]. These dams are mostly located upstream from the major carbon exporters in South America (pink triangles in Fig. 2f). If the new power plants were to break the sediment load linkage between the Amazon and the Andes, the migration rate of the big exporter of the Upstream Amazon basin would be greatly reduced, as also previously suggested by the Fluvial Dynamic Index[86]. Our analysis suggests a loss in carbon pumping equivalent to 4.2 ± 0.4 TgC/year (Table 1) if river morphodynamics will be totally halted. When these impacts are taken into account, the carbon intensity of the planned Peruvian and Bolivian dams (i.e., CO_2-equivalent emissions per unit of electricity generated) increases from 238 $gCO_{2eq}kWh^{-1}$[87] to 276 $gCO_{2eq}kWh^{-1}$, a value nearly three times higher than the United Nations Agenda 2030 recommendation for sustainable global electricity generation.

Table S1. Carbon fluxes reported in Fig. 1, with references. Colored rows refer to fluxes involved in the inland waters C-budget commented in the main text

Carbon flux	Reference	Role in Inland waters budget	Estimates (PgC/yr)	Level of Uncertainty*	Consequence on C cycle*
Outgassing	Raymond et al. (2013)<br>Drake et al. (2018)	Out-coming	2.1<br>3.9	high	high
Oceanic export	Cole et al. (2007)<br>Regnier et al. (2013)	Out-coming	0.9<br>0.95	low	low
Burial	Lal et. al (2003)<br>Battin et al.(2009)<br>Tranvik et al. (2009)<br>Aufdenkampe et al. (2011)<br>Regnier et.al (2013)	Out-coming	2.8-4.2<br>0.6<br>0.6<br>1.5<br>0.6	high	high
Autochthonous photosynthetic fixation	Regnier et al. (2013)	In-coming	0.3	high	moderate
Bed-rock weathering	Hartmann et al. (2009)	In-coming	0.5	-	-
C export from terrestrial ecosystem to inland water (resulting from a difference of the above fluxes)	Drake et al. (2018)	In-coming	2.9 - 6.7	high	high
Geological fluxes	Regnier et.al (2013)		0.15	-	
Fluxes from Atmosphere to Oceans and Estuaries (net) F_1	Regnier et.al (2013)**		2.1	-	-
Fluxes from Oceans and Estuaries to Sediments (net) F_2	Regnier et.al (2013)***		0.65	-	-
Silicate and Carbonate weathering F_3	Hartmann et al (2009)<br>Regnier et.al (2013)		0.3-0.44<br>0.35	-<br>-	 -
Antropogenic fossil-fuel emissions F_4	Regnier et.al (2013)		7.9	-	-

*According to Drake et al. (2018). ** Budget of CO_2 emissions and uptake from estuaries and air-sea CO_2 flux in the Oceans. *** Total C burial in estuarine and oceans sediment. References: Raymond et al. (2013)[18]; Drake et al. (2018)[7]; Cole et al. (2007) [1]; Regnier et al. (2013)[19]; Lal et al. (2003)[17]; Battin et al. (2009)[4]; Tranvik et al. (2009)Tranvik[31]; Aufdenkampe et al. (2011), Hartmann et al.(2009)[20];

Table S2. List of the global databases analysed in the present work. *Dataset updated annually, version 1.7 was used in this study which analyzes the period 2000-2019. **Dataset updated annually, version 1.3 was used in this study which analyzes the period 2000-2019. References: Baccini et al. (2012)[44]; Hansen et al. (2013), [71]; Pekel et al. (2016)[70]; USGS[75] ; Linard et al. (2012)[88]; Gaughan et al. (2013)[89]; Sorighetta et al. (2015)[90]; Biosphere Programme classification (IGBP)[77]; et al. (2019)[32]; Linke et al. (2019)[72].

	Dataset	Description	Data source	Use in this paper (Section)
1)	WHRC Carbon Stock	A national-level map of above-ground live woody biomass density for tropical countries at 500m resolution. This dataset was assembled from a combination of co-located field measurements, LiDAR observations, and imagery recorded from the Moderate Resolution Imaging Spectroradiometer (MODIS).	Baccini (2012)	Estimation of carbon density within regions of interest (EMCE Computation - Method M4), Calibration of model for plant growth (SI-Logistic growth model – Method M2), (Classification of the biomass distribution signature).
2)	WHRC Above-ground Live Woody Biomass Density	Global-scale, map of aboveground biomass (AGB) at approximately 30-meter resolution. This data product expands on the methodology presented in Baccini et al. (2012) to generate a global map of aboveground live woody biomass density (megagrams biomass ha-1) for the year 2000.	Zarin (2016)	Estimation of carbon density within regions of interest (EMCE Computation – Methods M1, M2, M3), Calibration of model for plant growth (SI-Logistic growth model- Method M2).
3)	Global Forest Change	Results from time-series analysis of Landsat images to characterize global forest extent and change.	Hansen (2013)* dataset version 1.7 (2000-2019)	Identification of river-driven forest loss RDFL (River selection and data filtering).
4)	Global Surface Water	Maps of the location and temporal distribution of surface water from 1984 to 2019 and statistics on the extent and changes of those water surfaces.	Pekel (2016)** dataset version 1.2 (2000-2019)	Assessment of wet area within regions of interest (ROIs definition).
5)	MODIS Burned Area Monthly Global 500m	The Terra and Aqua combined MCD64A1 Version 6 Burned Area data product is a monthly, global gridded 500m resolution product containing per-pixel burned-area and quality information.	USGS (2000-2019)	Definition of probability map of river-driven forest loss loss $P_{j,k}^{(wf)}$ (River selection and data Filtering).
6)	WorldPop Global Project Population Data	Global high-resolution, contemporary data on human population distributions.	Linard (2012) Gaughan (2013) Sorichetta (2015)	Definition of probability map of river-driven forest loss loss $P_{j,k}^{(u)}$ (River selection and data Filtering).
7)	MODIS Land Cover Type Yearly Global 500m	The MCD12Q1 V6 product provides global land cover types at yearly intervals (2001–2019) derived from six different classification schemes.	Biosphere Programme classification (IGBP)	Definition of probability map of river-driven forest loss $P_{j,k}^{(lc)}$ (River selection and data filtering).
8)	Free Flowing Rivers	Mapping the world's free-flowing rivers.	Grill (2019)	Identification of natural river reaches not impacted by human activities CSI (River selection and data filtering).
9a) 9b)	HydroATLAS RiverATLAS	Comprehensive database presenting a wide range of hydro-environmental attributes from existing global datasets in a consistent and organized manner.	Linke (2019)	Assessment of Strahler index of river reaches (River selection and data filtering) and downscaling (Downscaling).

Table S3. Results of the classification algorithm and partition of the carbon signature at continental scale. NS: negatively skewed; MM: multi-modal; PS: positively skewed; BS: bell-shaped.

	NS	MM	PS	BS
**Catchment scale**				
Upstream Amazon Basin	66.7 %	22.2 %	11.1 %	0 %
Central Amazon Basin	60.0 %	30.7 %	5.3 %	4.0 %
Downstream Amazon Basin	27.4 %	45.4 %	15.1 %	12.1 %
Others	18.5 %	29.6 %	38.9 %	13.0 %
**Continental scale**				
Neotropics	47.9 %	29.9 %	16.2 %	6.0 %

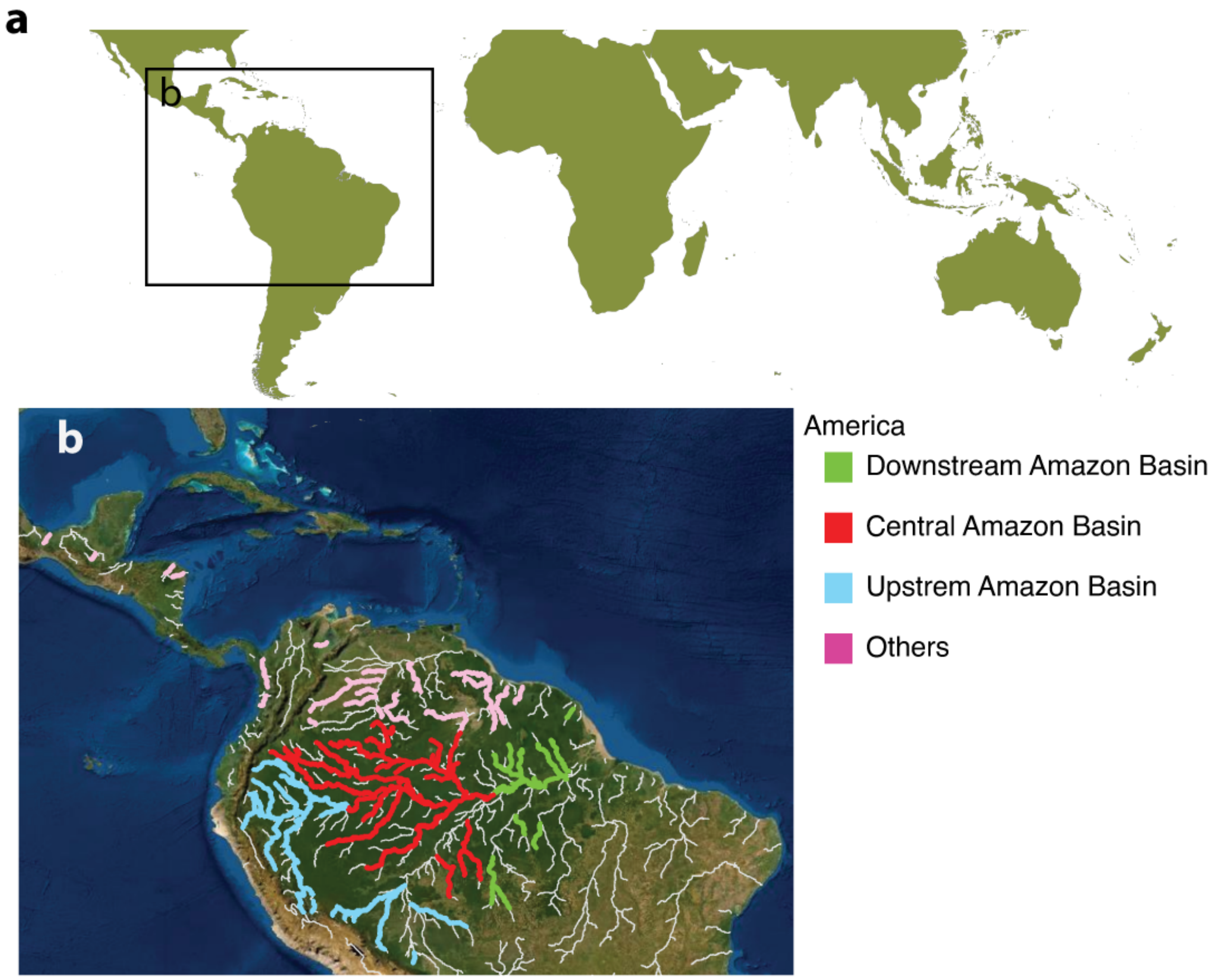

Figure S1. Zonation of river basins

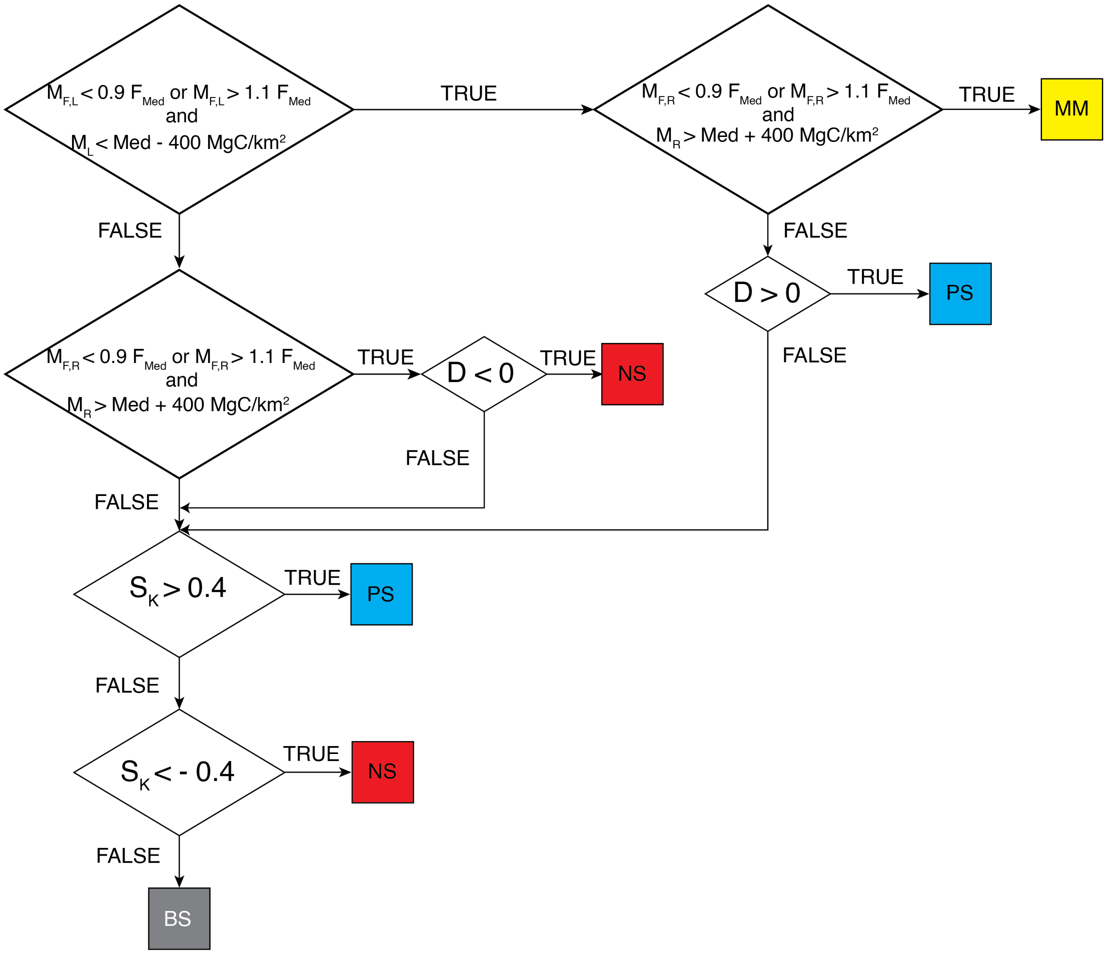

Figure S2. **Classification Algorithm**

Table S4. IGBP Classification

Classes	IGBP (Land Cover – Type1)
Natural with High vegetation density (NHV)	Evergreen Needleleaf forest<br>Deciduous Needleleaf forest<br>Evergreen Broadleaf forest<br>Deciduous Broadleaf forest<br>Mixed Forest<br>Closed/Open Shrublands<br>Grasslands and Permanent Wetlands
Natural with Low vegetation density (NLV)	Savannas<br>Woody Savanna
Anthropic (AN)	Croplands<br>Croplands/Natural vegetation mosaics<br>Urban and Built-up lands
Unvegetated (UV)	Water bodies<br>Permanent snow and ice<br>Barren

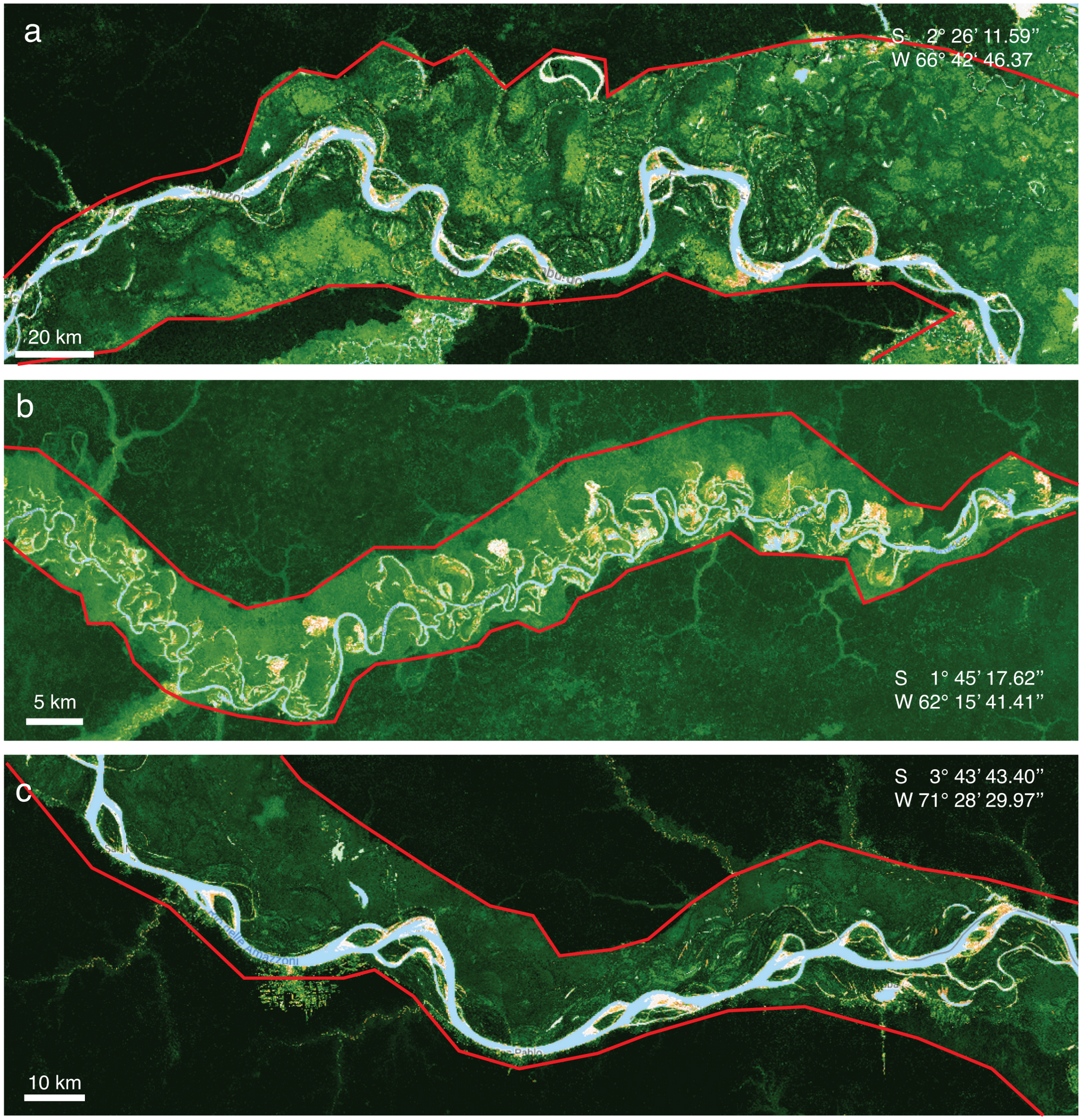

Figure S3. Three examples of carbon density map, from dataset n.2[78] of Supplementary Table S2, and the lateral extension of ROIs (red solid line). The recurrent fluvial disturbances that affect floodplain vegetation and promote a continuous rejuvenation of riparian corridors (removal of mature vegetation and colonization by seedlings and young trees of bare riverbanks) thus inducing an immature stage and a lower C-stock than non-flooded mature forests[50,85]. An edge between carbon distributions of the disturbed floodplains and tierra ferma is well visible in the carbon map developed by ref.[78] and it was used to define the lateral extension of ROI (red line). (a) Amazon river, near the Jutai river confluence; (b) Rio Cuiuni (c) The upper Amazon River near Iquitos (Perù).

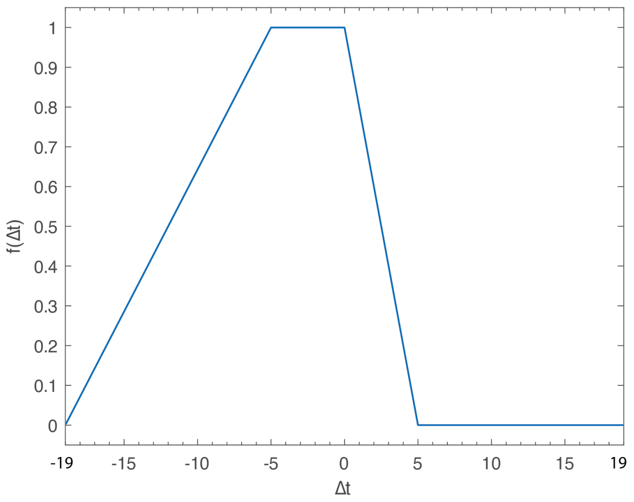

Figure S4. **The function f (Δt) mentioned in the Probabilistic Classification model.**

Table S5. Results about the aggregated continental eCE (by using the methods M1-M4) and the corresponding uncertainties, for the largest tropical free flowing rivers (width >200 m.)

Methods Methods	eCE (TgC/yr) **America**	Autocorrelation scale	Carbon uncertainties	eCE uncertainties (TgC/yr) **America**
M1	8.60	500 m	50% $\rho_{j,k}$	0.06
			75% $\rho_{j,k}$	0.09
			100% $\rho_{j,k}$	0.12
			125% $\rho_{j,k}$	0.15
		ROI scale	50% $\rho_{j,k}$	0.39
			75% $\rho_{j,k}$	0.51
			100% $\rho_{j,k}$	0.67
			125% $\rho_{j,k}$	0.84
M2	8.89	500 m	50% $\rho_{j,k}$	0.06
			75% $\rho_{j,k}$	0.09
			100% $\rho_{j,k}$	0.12
			125% $\rho_{j,k}$	0.15
		ROI scale	50% $\rho_{j,k}$	0.39
			75% $\rho_{j,k}$	0.51
			100% $\rho_{j,k}$	0.67
			125% $\rho_{j,k}$	0.84
M3	7.91	ROI scale	Spatial St.Dev. ρ	0.22
M4	8.62	ROI scale	Spatial St.Dev. ρ	0.23

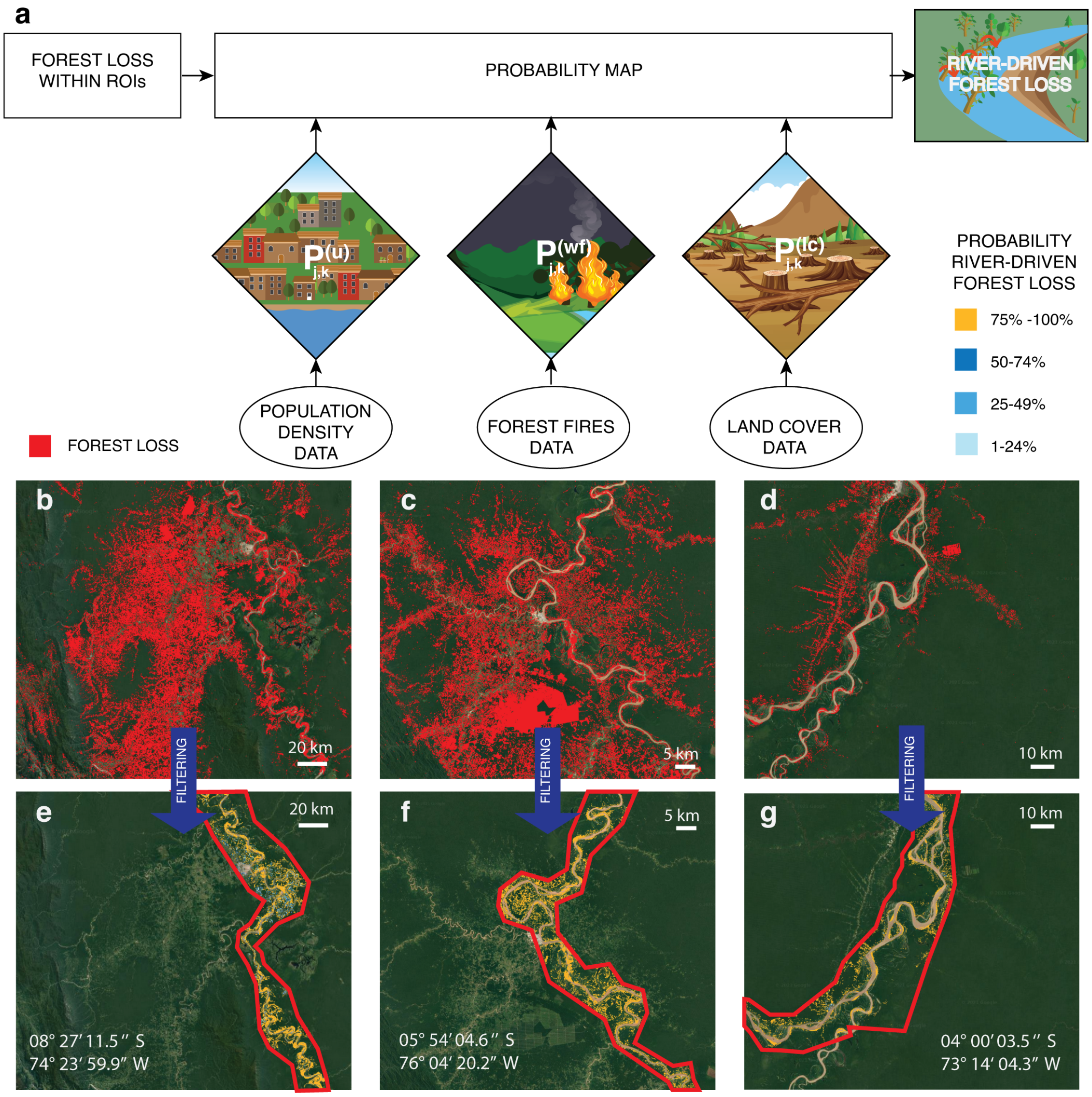

Figure S5. Filtering procedure. (a) Conceptual algorithm. (b-d) Satellite images before filtering, with forest loss reported in red. (e-g) Same images after filtering, with the probability of River-Driven Forest Loss clustered in four classes (see legend). Thick red lines refer to the ROI boundaries.

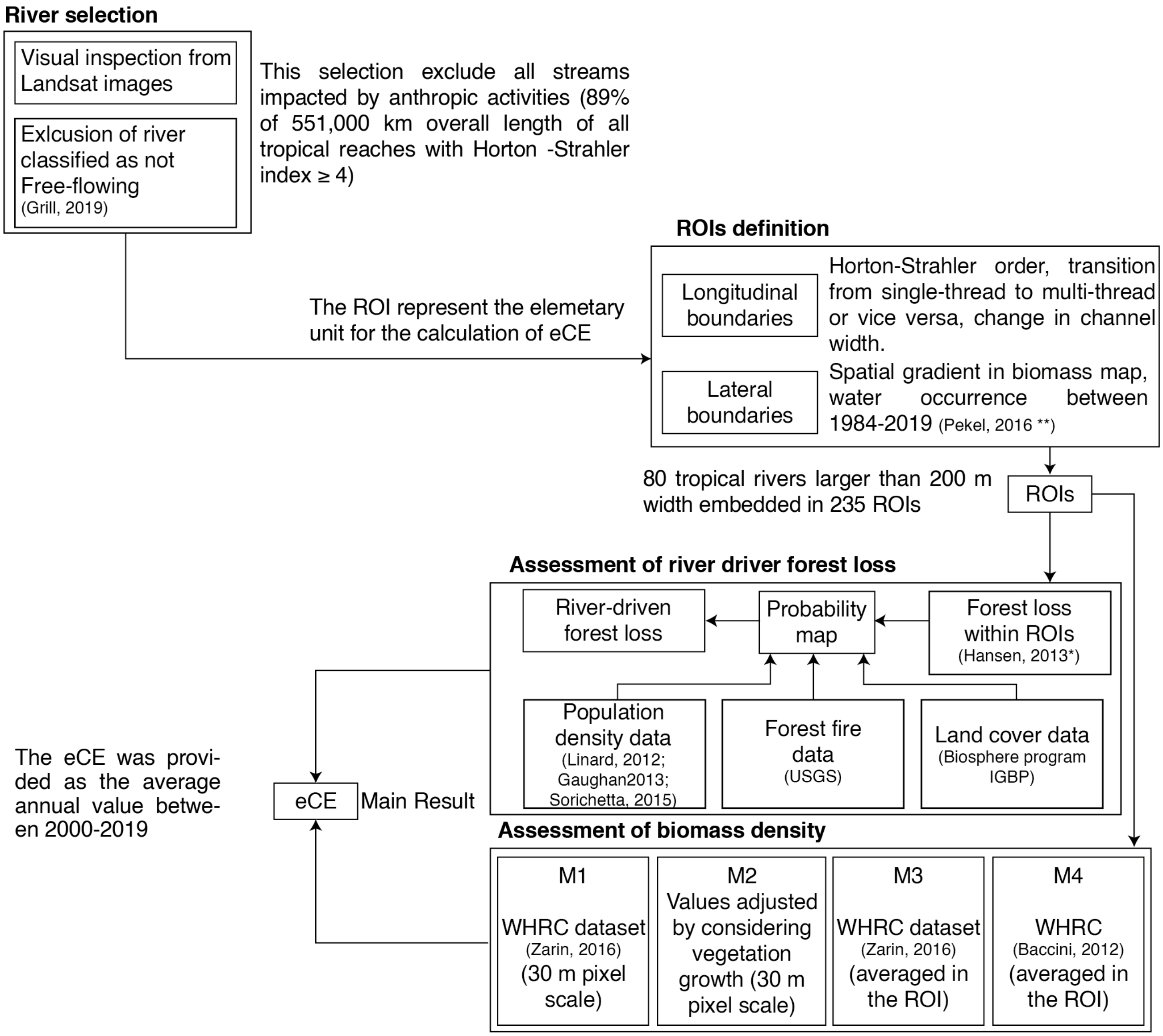

Figure S6. Recapitulation of the procedure for the eCE assessment. For further details on the datasets used refer to Table S2. *Dataset updated annually; version 1.7 was used in this study which analyzes the period 2000-2019. **Dataset updated annually; version 1.3 was used in this study which analyzes the period 2000-2019. See also the userguide.pdf file deposited in the Online Material (Figshare Repository).

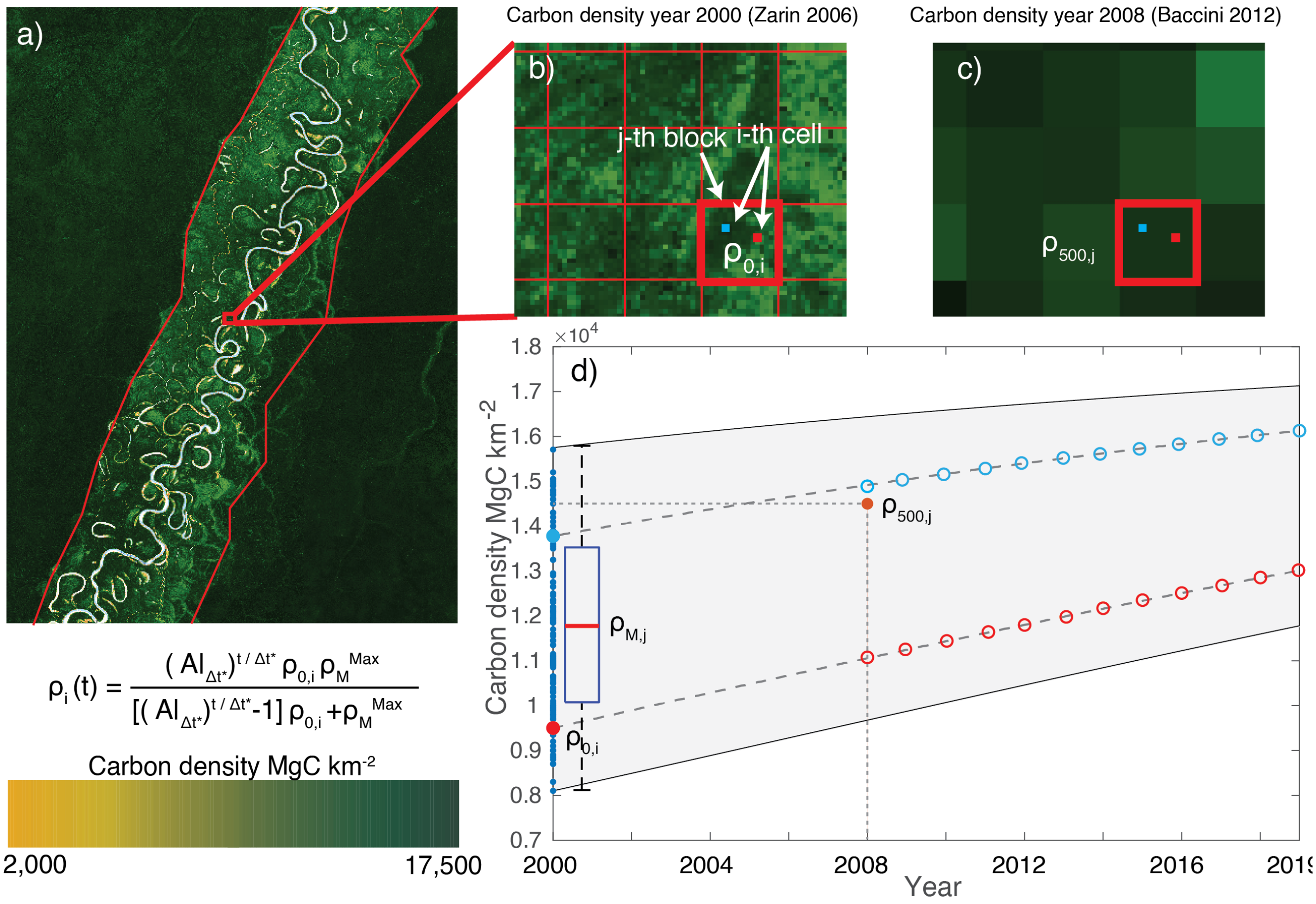

Figure S7. **Application of the logistic growth update developed in Methods M2, through the use of equations (11) and (13).** For each ROI (a) the value of carbon density of the generic pixel *i* was corrected in order to account the vegetation growth between the acquisition time (b) and the time of the forest loss (if occurred after 2008), through the equation (13). The model was calibrated by comparing the carbon biomasses reported by two different datasets with different acquisition times: i) dataset from ref. Ref.[78] acquired in 2000 (panel B); ii) dataset from ref.[44] acquired 2008 (panel c). In the case the forest loss occurred before 2008, the value of carbon density was not updated. An example of result for the extrapolated annual values of carbon density for two generic pixels are reported in panel d (blue and red open points), whereas the blue close points and the boxplot refer to the values of all cells within the j-th block at t=2000.

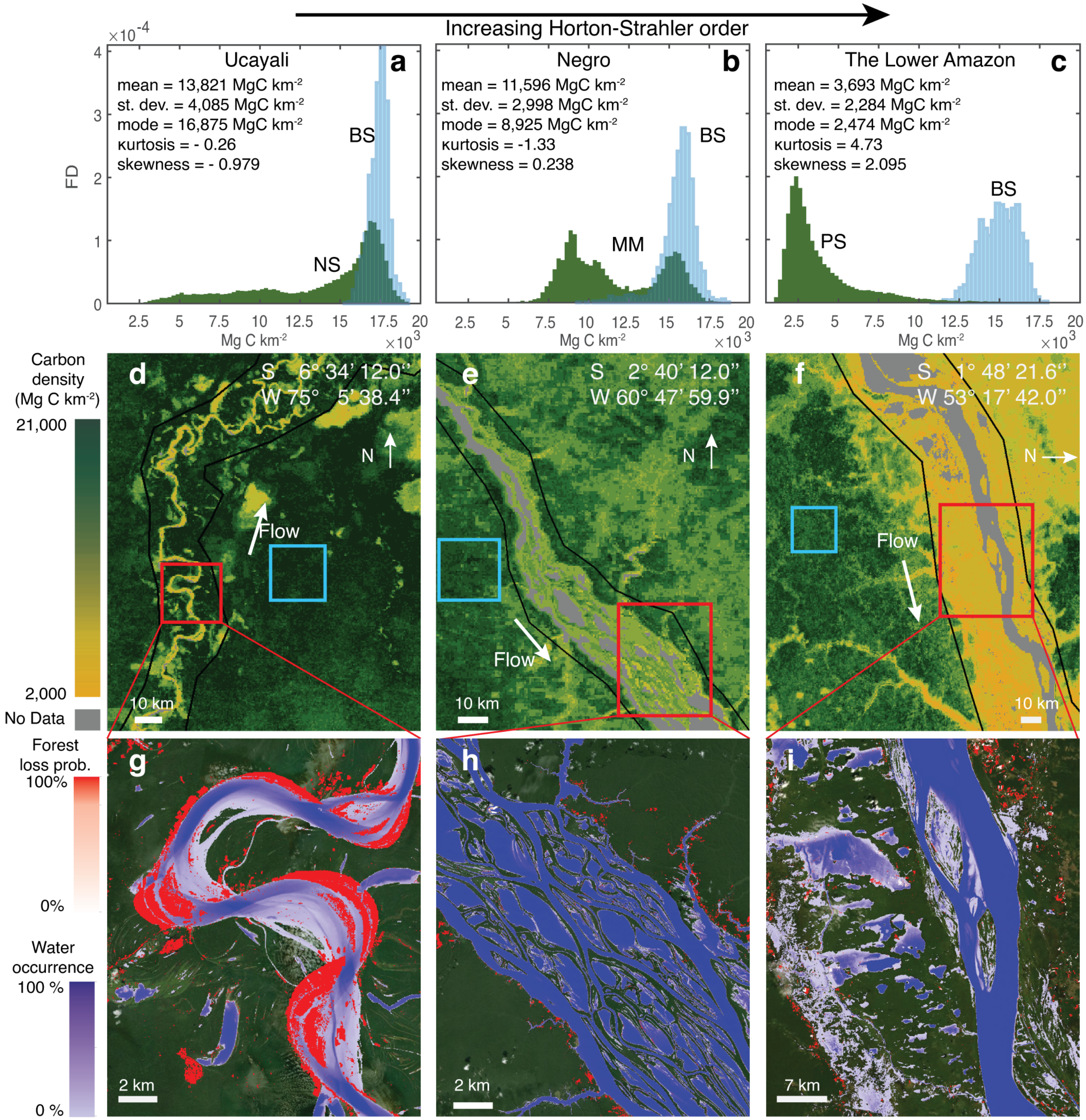

Figure S8. **Signature of planforms on carbon density distributions for a meandering river (Ucayali, left column), and two multi-thread rivers, the Negro River (middle column) and the Lower Amazon (right column).** (a-c) Frequency distribution of biomass carbon density. Green histograms refer to the entire ROIs reported in panels d-f (black polygons), blue histograms refer only to the region in the blue box (i.e., where river dynamics do not affect vegetation). NS: negatively skewed; MM: multi-modal; PS: positively skewed; BS: bell-shaped. (d-f) Maps of carbon density distribution for corresponding regions based on dataset by (43); (g-i) River-driven forest loss probability P (red) over 2000-2019 (see Sect. Probabilistic Classification Model) and water surface occurrence (blue) from the dataset by ref.[70].

Legend Suppementary Tables (spreadsheet)

S6 AllResults.xlxs This dataset contains computational results of Geomorphic carbon pumping (eCE Methods M1-M4, eCE_A, A_{RDFL}, uncertainty parameters), carbon signature classification and statistical parameters of carbon density distributions, polygon coordinates and feature (area) of each ROI and length of reaches of order n (1-10) within ROI.

S7 NoFreeFlowRiversRESULTS.xlsx This dataset contains computational results of eCE for impacted rivers.

References

86. Latrubesse, E. M. *et al.* Damming the rivers of the amazon basin. *Nature* **546**, 363–369 (2017).

87. Almeida, R. M. *et al.* Reducing greenhouse gas emissions of Amazon hydropower with strategic dam planning. *Nat. Commun.* **10**, 4281, DOI: 10.1038/s41467-019-12179-5 (2019).

88. Linard, C., Gilbert, M., Snow, R. W., Noor, A. M. & Tatem, A. J. Population distribution, settlement patterns and accessibility across Africa in 2010. *PLoS ONE* **7**, DOI: 10.1371/journal.pone.0031743 (2012).

89. Gaughan, A. E., Stevens, F. R., Linard, C., Jia, P. & Tatem, A. J. High resolution population distribution maps for Southeast Asia in 2010 and 2015. *PloS one* **8** (2013).

90. Sorichetta, A. *et al.* High-resolution gridded population datasets for Latin America and the Caribbean in 2010, 2015, and 2020. *Sci. data* **2**, 1–12 (2015).
